# Amorphous NdIZO Thin Film Transistors with Contact-Resistance-Adjustable Cu S/D Electrodes

**DOI:** 10.3390/membranes11050337

**Published:** 2021-04-30

**Authors:** Xinyi Zhang, Kuankuan Lu, Zhuohui Xu, Honglong Ning, Zimian Lin, Tian Qiu, Zhao Yang, Xuan Zeng, Rihui Yao, Junbiao Peng

**Affiliations:** 1 Institute of Polymer Optoelectronic Materials and Devices, State Key Laboratory of Luminescent Materials and Devices, South China University of Technology, Guangzhou 510640, China; mszhangx1@mail.scut.edu.cn (X.Z.); mskk-lu@mail.scut.edu.cn (K.L.); 201730321193@mail.scut.edu.com (Z.L.); yangzhao@china-fenghua.com (Z.Y.); 201766303200@mail.scut.edu.cn (X.Z.); psjbpeng@scut.edu.cn (J.P.); 2 Guangxi Key Lab of Agricultural Resources Chemistry and Biotechnology, Yulin Normal University, Yulin 537000, China; xzh21@ylu.edu.cn; 3 Department of Intelligent Manufacturing, Wuyi University, Jiangmen 529020, China; qiutian@ustc.edu; 4 State Key Laboratory of Advanced Materials and Electronic Components, Fenghua Electronic Industrial Park, No. 18 Fenghua Road, Zhaoqing 526020, China

**Keywords:** thin film transistors, contact resistance, copper electrode, NdIZO

## Abstract

High-performance amorphous oxide semiconductor thin film transistors (AOS-TFT) with copper (Cu) electrodes are of great significance for next-generation large-size, high-refresh rate and high-resolution panel display technology. In this work, using rare earth dopant, neodymium-doped indium-zinc-oxide (NdIZO) film was optimized as the active layer of TFT with Cu source and drain (S/D) electrodes. Under the guidance of the Taguchi orthogonal design method from Minitab software, the semiconductor characteristics were evaluated by microwave photoconductivity decay (μ-PCD) measurement. The results show that moderate oxygen concentration (~5%), low sputtering pressure (≤5 mTorr) and annealing temperature (≤300 °C) are conducive to reducing the shallow localized states of NdIZO film. The optimized annealing temperature of this device configuration is as low as 250 °C, and the contact resistance (R_C)_ is modulated by gate voltage (V_G_) instead of a constant value when annealed at 300 °C. It is believed that the adjustable R_C_ with V_G_ is the key to keeping both high mobility and compensation of the threshold voltage (V_th_). The optimal device performance was obtained at 250 °C with an I_on_/I_off_ ratio of 2.89 × 10^7^, a saturation mobility (μ_sat_) of 24.48 cm^2^/(V·s) and V_th_ of 2.32 V.

## 1. Introduction

Amorphous oxide semiconductors (AOS) are widely used as the active layer of high performance and flexible thin film transistors (TFTs) [1] because of their high mobility, uniformity and the insensitivity of their electrical properties to mechanical strain. Gallium-doped indium-zinc-oxide (In-Ga-Zn-O, IGZO), which is now one of the most essential channel materials for amorphous oxide semiconductor (AOS)-TFTs, was first fabricated by Nomura and Hosono in 2004 [2]. It has been proven to have advantages such as high mobility [3], good uniformity [4], visible light transparency [4], low processing temperature [5] and low cost, but it still has limitations such as relatively low mobility and high annealing temperature [6,7,8]. Therefore, new, effective dopants need to be developed to take the place of Ga without significantly decreasing the mobility of IZO. Research has demonstrated that doping neodymium (Nd) atoms into IZO can attract electrons and inhibit the generation of excess carriers resulting from oxygen vacancy (V_O_) [9] more effectively due to its low electronegativity (Nd = 1.1) and high oxygen bond dissociation energy (703 kJ/mol) in comparison with the other above dopants [10,11,12]. Furthermore, Nd_2_O_3_ and In_2_O_3_ share the same bixbyite structure, which may result in fewer defects [13].

On the other hand, with displays with high resolution (≥8 K), high frame-rate (≥480 Hz) and large size (≥110 inches) becoming increasingly popular, copper (Cu) is considered to be the most promising electrode material in TFTs due to its low resistivity, good thermal stability, high thermal conductivity and good electromigration reliability [14,15]. As the source and drain (S/D) electrodes have direct contact with the semiconductor layer, the contact characteristics between them are very important in the research of TFTs with Cu S/D electrodes. However, the above-mentioned research has only focused on the active layer of NdIZO-TFTs, and the contact conditions between Cu S/D electrodes and the NdIZO layer still need to be investigated in detail. The contact resistance (R_C_) of TFT specifically refers to the interface resistance arising from the metal–semiconductor contact, and the value of R_C_ will directly affect the mobility of TFT [16]. When the R_C_ is comparatively large, the transport efficiency of carriers is limited, resulting in low mobility and affecting the electrical performance of the device. Due to the advancement in micro-nano manufacturing technology, the pursuit for higher carrier concentration and shorter channel length (L) is inevitable. This increases the influence of the R_C_ on the electrical properties of the metal oxide thin film transistor, such as carrier mobility [17]. Saturation mobility (μ_sat_) is often presumed to be free of contact effects, which is reasonable at sufficiently high V_GS_-V_T_. However, this has been shown to be incorrect for high contact resistance, especially at the point of the turn-on, for the presence of a Schottky barrier, and where nontrivial Rc causes gated contact resistance [18,19]. So, it is essential to study the R_C_ of NdIZO-TFTs with Cu S/D electrodes.

This paper demonstrated the influence of manufacturing parameters on the semiconductor characteristics of the NdIZO layer by μ-PCD measurement. In addition, Cu S/D electrodes were utilized in the device with an optimized NdIZO layer, and the interface contact was investigated by the transmission line method (TLM) method.

## 2. Materials and Methods

**Figure 1:**
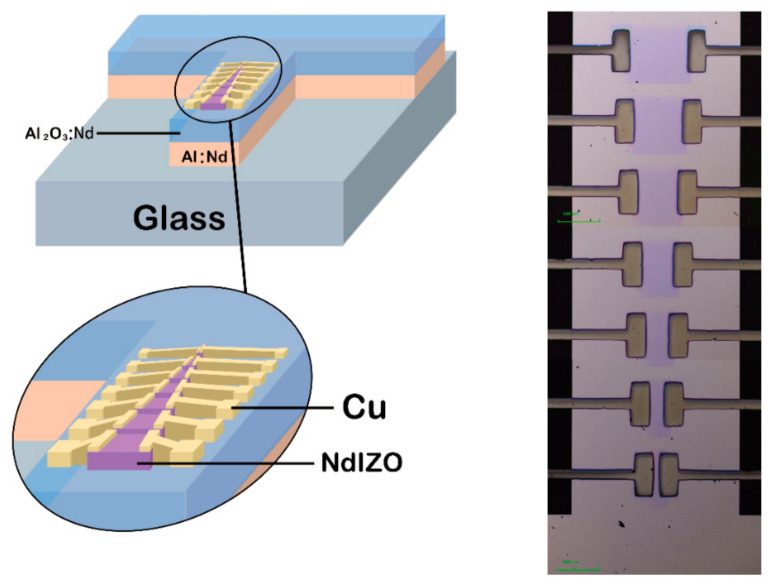
The structure of NdIZO-TFT.

The structure of the vacuum sputtering thin film transistor is shown in Figure 1, and the specific description is as follows.

A 300 nm Al-Nd alloy (Al:Nd) film was deposited on a glass substrate using physical vapor deposition (PVD) by direct current (DC) magnetron sputtering, and then formed into a T-shape pattern as the gate by wet etching. The anodization was performed in a mixed solution of ethylene glycol and ammonium tartrate. A constant current of 0.1 mA/cm^2^ was applied to the layer of the Al:Nd gate electrode prepared as above until the desired voltage (100 V) was reached. Then, the final voltage (100 V) was kept on for 1 h to form a 200 nm Al_2_O_3_:Nd film as a gate insulating layer. The array prepared with the Al_2_O_3_:Nd insulating layer was ultrasonically cleaned with deionized water for 15 min and with isopropanol for another 15 min. The sample was dried in an oven at an ambient temperature of 80 °C. Then, the prepared array was placed on the metal mask plate and deposited a 15 nm NdIZO film by radio frequency (RF) magnetron sputtering. The NdIZO target used had a diameter of 5.08 cm and a composition of Nd_2_O_3_:In_2_O_3_:ZnO = 1:62.5:36.5 (wt. %). Subsequently, the sample was annealed at a certain temperature for 1 h to eliminate defects in the film and improve its electrical properties. The oxygen concentrations during RF magnetron sputtering were 0%, 5% and 10%, the sputtering pressures applied were 3.38 mTorr, 5 mTorr and 8 mTorr, and the chosen annealing temperatures were 25 °C, 200 °C, 250 °C, 300 °C, 350 °C and 400 °C. As the full factor experiment requires excessive amount of time and consumables, this work used the Taguchi orthogonal design method from Minitab software and finally selected 18 representative combinations for the measurement and analysis of experimental data. After sputtering the active layer under appropriate conditions, a Cu film of about 300 nm was deposited as S/D electrodes by RF magnetron sputtering. The Cu target used had a diameter of 5.08 cm and its composition was pure Cu. The sputtering conditions included power of 100 W and atmospheric pressure of 3 mTorr. Both the sputtering of the active layer and the S/D electrodes used the metal mask plate to achieve patterning.

**Figure 2:**
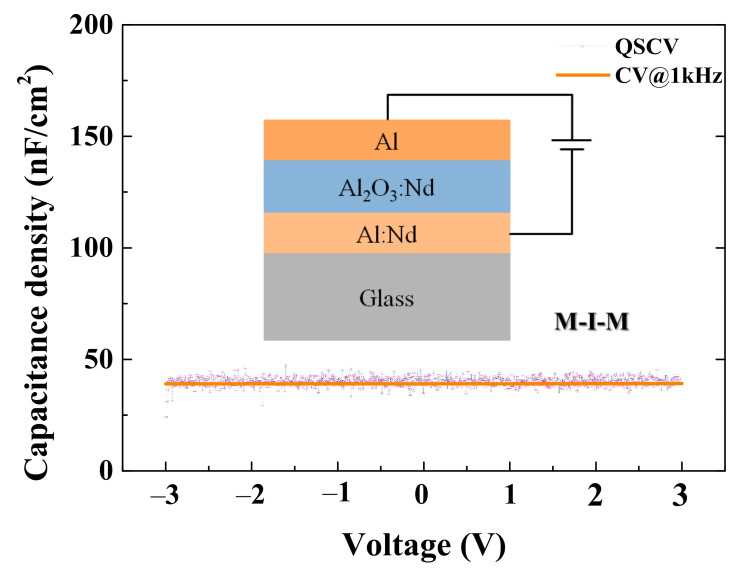
Quasi-static and 1 kHz CV characteristic of the M-I-M capacitor.

An metal-insulator-metal (M-I-M) capacitor corresponding to the NdIZO-TFT on silicon substrate was fabricated, and the measured C_i_ in the frequency range below 10 Hz was 42.02 ± 2.07 nF/cm^2^ while C_i_ at 1 kHz was around 39.07 nF/cm^2^, as shown in Figure 2. X-ray diffraction (XRD) was utilized to determine the phases of thin films using Cu Kα1 radiation (*λ* = 0.15418 nm). XRD measurement was performed by Empyrean Nano edition (Empyrean Nano edition, PANalytical, Almelo, The Netherlands). X-ray photoelectron spectroscopy (XPS) analysis was carried out to investigate the chemical changes in the oxide films by using a THERMO ESCALAB250Xi (Thermo Fisher Scientific, Waltham, MA, USA) with an Al Ka (hν = 1486.6 eV) 15 kW beam spot source. Microwave photoconductivity decay (μ-PCD, LTA-1620SP) was used to characterize the carrier decay characteristics of the film, and Minitab was applied to conduct data analysis and select the optimal growing conditions for the film. A semiconductor analyzer was used to characterize the electrical performance of the device. The transfer curve measured when V_D_ = 0.1 V along with the TLM was used to evaluate the contact performance of the electrode. Through calculation, the total resistance (R_total_) of the TFT of each channel length could be obtained. Then, taking R_total_ as the *y*-axis and channel length as the *x*-axis, the relationship between R_total_ and L was linearly fitted. The slope of the fitted line is the total channel resistance (r_ch_), and the intersection on the *y*-axis is the contact resistance (R_C_).

## 3. Results and Discussion

### 3.1. Film Deposition

**Figure 3:**
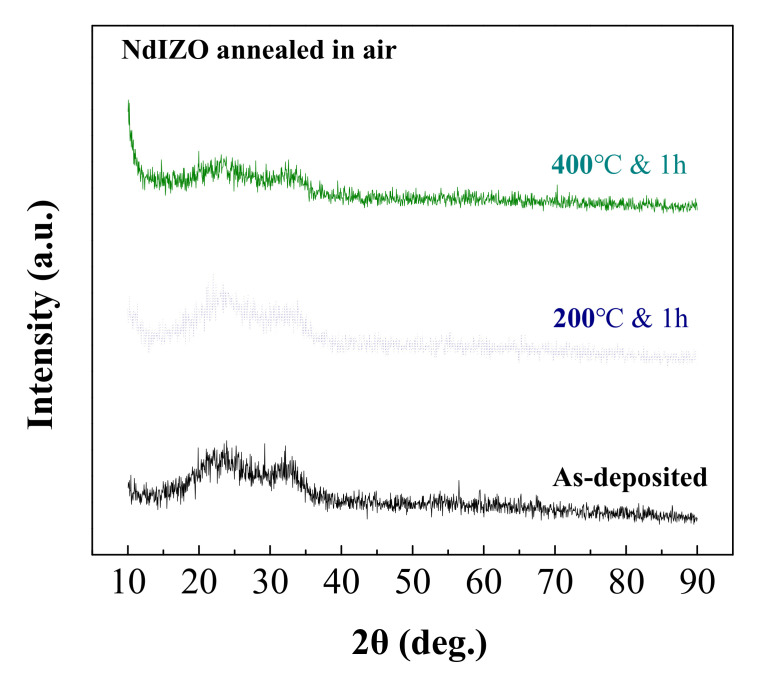
XRD pattern of NdIZO films with different annealing temperatures.

Figure 3 shows the XRD of the thicker NdIZO films (with a thickness of 40–50 nm) with increasing annealing temperature. The spectra indicate that the as-deposited films were amorphous and even remained amorphous after annealing at 400 °C for 1 h in air. Besides, two broad peaks between 20° and 35° were found due to the glass substrate [20].

The μ-PCD method as a non-contact and non-destructive technology with low cost and short processing time [21] was used to measure the sub-gap states of the films and the decay of carriers in the film under different deposition conditions. In the μ-PCD measurement, laser irradiation activated excess carriers and the density of the carriers obeys Equation (1) [22].

n(t) = n_0_{exp(−t/τ_1_) + exp [−(t/τ_2_)^β]} (1)

where n_0_ is the carrier density after laser irradiation, τ_1_ and τ_2_ are fast and slow decay constants, and β is the stretching exponent.

**Figure 4:**
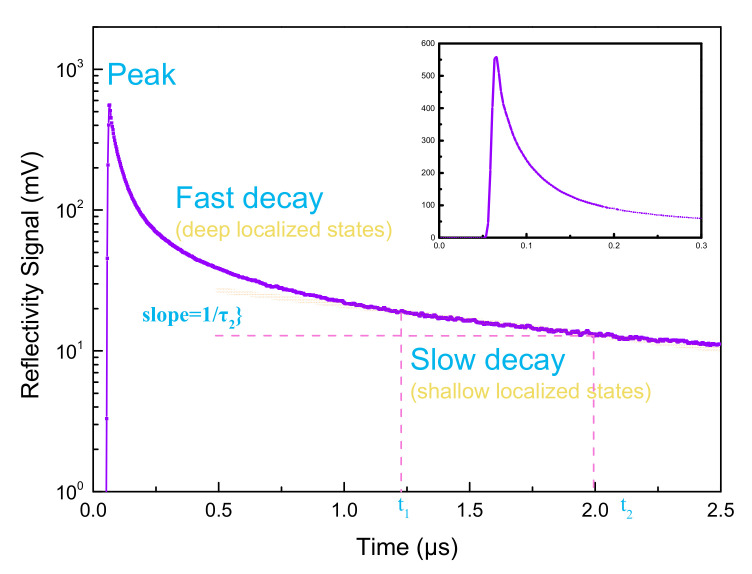
Photoconductivity response for NdIZO film annealed at 200 °C.

Figure 4 shows the fitted curve of a portion of carriers measured by μ-PCD using Equation (1).

The decay curve can be divided into three components: peak value, fast decay and slow decay. The peak value, which is related to the density of the conduction band tail, originates from the recombination process of photon-generated carriers during the laser pulse irradiation. The fast decay indicates the rapid recombination of the photon-generated carriers, which is related to the recombination process through the deep localized state. The slow decay is attributed to the density of the photonic band gap state, which is assumed to be related to the trapping process of volume defects and other factors [23]. However, fast decay has a short lifetime and is difficult to observe directly. When the pulse width of the laser is large enough relative to the lifetime, the peak is proportional to the lifetime. Therefore, for the evaluation of deep level traps, μ-PCD uses peak values that can be measured quickly and accurately, rather than using the lifetime value of rapid decay [24]. So, the two characteristic parameters obtained by analyzing the μ-PCD decay curve are the peak value and τ_2_, where the peak value represents the number of carriers and τ_2_ is related to film uniformity. The slow decay τ_2_ corresponds to the slope between t_1_ and t_2_, as shown in Figure 4 [25]. The higher the peak value and the lower τ_2_, the better the quality of the film.

In order to save experimental costs, this work used the Taguchi orthogonal design method to create an L18 orthogonal array experiment [26,27] with 3 parameter elements, one at six levels and two at three levels, as shown in Table 1 with their corresponding peak value and τ_2_.

**Figure 5:**
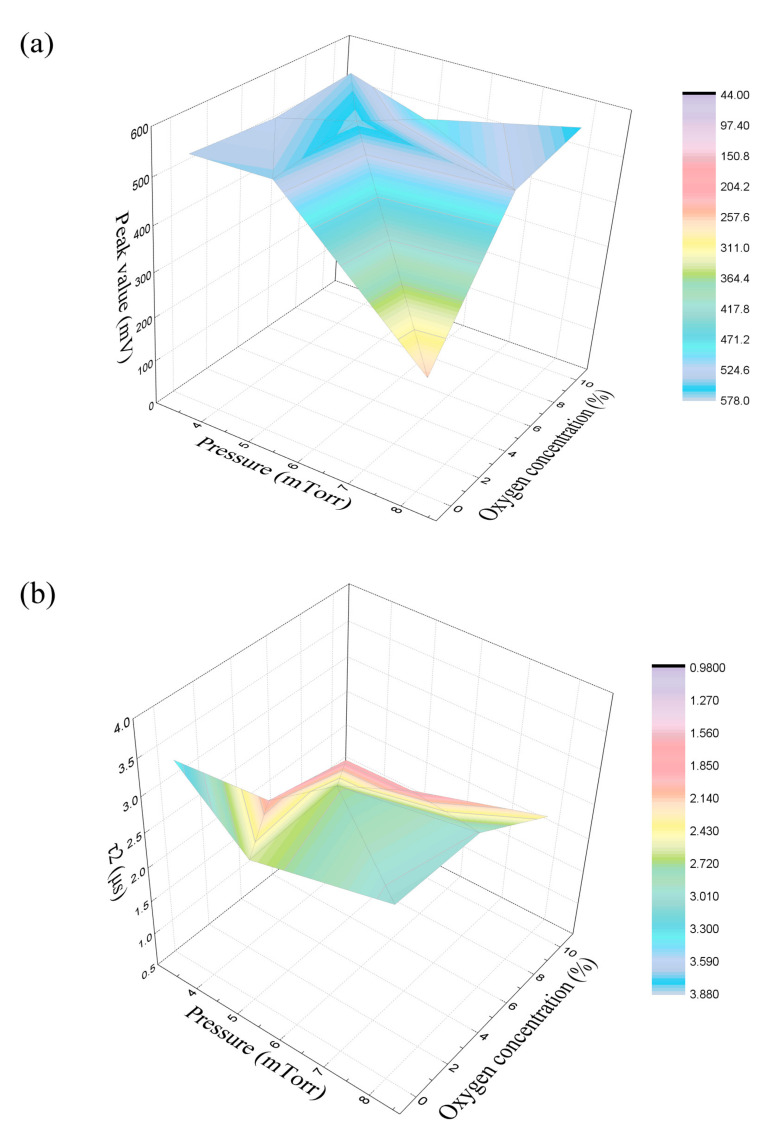
The influence of oxygen concentration and sputtering pressure on the μ-PCD parameters. (**a**) Peak value, (**b**) τ_2_.

Figure 5 shows the relationship between the peak value and τ_2,_ and oxygen concentration and sputtering pressure. From Figure 5a, it can be seen that the curved surface reaches its peak when the pressure is 3.5–5.5 mTorr and the oxygen concentration is between 4% and 8%. From Figure 5b, it can be seen that when the pressure rises from 3.38 mTorr to 8 mTorr with low oxygen concentration, τ_2_ decreases rapidly at first and then increases slowly, and reaches a minimum value of around 4.5 mTorr. τ_2_ shows a roughly increasing trend with the increase in the pressure at high oxygen concentration. With the increase in the oxygen concentration, τ_2_ decreases sharply at first, then tends to be flat, and then decreases slowly. This may be because when the oxygen concentration is low, with the decrease in oxygen concentration during sputtering, the concentration of carrier in the film increases due to the increase in donor-like defects. High oxygen concentration will lead to the reduction of oxygen vacancies, which mainly provide the carriers in oxide semiconductors [28]. This change results in a corresponding reduction in carrier concentration and τ_2_.

**Figure 6:**
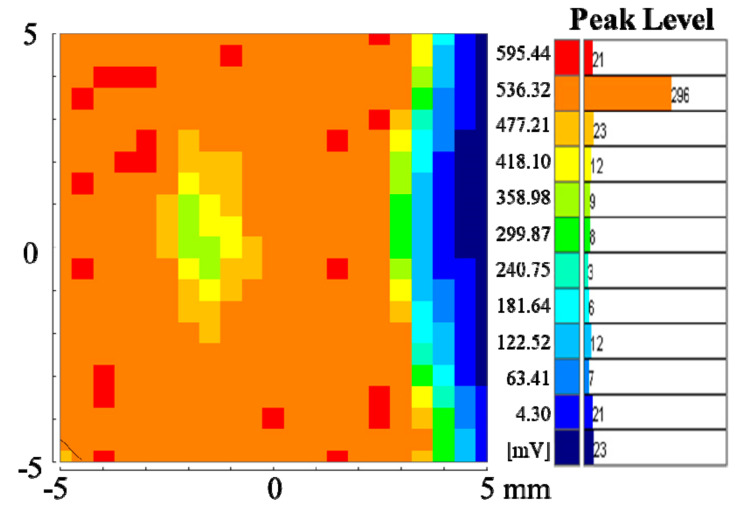
The μ-PCD mapping scan result with pressure of 5 mTorr and oxygen concentration of 5%.

Based on the above analysis, in order to balance the peak value and τ_2_, the final choice for the sputtering condition of the active layer was: 5 mTorr and 5% of oxygen. The appropriate peak value was the one that would not generate excess carriers at the above condition. Figure 6 shows the μ-PCD mapping scan result with a scanning area of 0.5 * 0.5 mm for each point [29]. The performance of the carriers and their distribution in the film were considered to be uniform [30], which is consistent with a low τ_2_.

The empirical formulae representing the weight of factors acting on target objects can be also obtained using the Taguchi orthogonal design method of Minitab software [31,32]. During the experiment, we also chose the annealing temperature as a factor that affects the peak value and τ_2_. The formulas for peak value and τ2 with oxygen concentration, sputtering pressure and annealing temperature are as follows in Equations (2) and (3).

Peak value (mV) = 675.0 − 20.3 Pressure (mTorr) + 8.29 Oxygen concentration (%) − 0.396 Annealing temperature (°C)(2)

τ_2_ (μs) = 2.714 + 0.0980 Pressure (mTorr) − 0.1105 Oxygen concentration (%) − 0.00075 Annealing temperature (°C) (3)

Obviously, the impact of the annealing temperature on peak value and τ_2_ is much lower than that of sputtering oxygen content and sputtering pressure. It can be generally seen that the film uniformity and carrier mobility around 200–300 °C are good, but the device optimization needs to be further studied. In order to ensure the accuracy of the experiment, the annealing temperature should still be used as a variable in the follow-up preparation of the device, and further study could be conducted on its influence on the threshold voltage, contact resistance and other electrical performance parameters of the thin film transistor.

### 3.2. Thin Film Transistors

In order to further explore the electrical properties of the NdIZO film, a semiconductor analyzer was used to measure the transfer and output curves of the prepared thin film transistors, and several formulas were used to calculate the I_on_/I_off_ ratio, saturation mobility, subthreshold swing, threshold voltage, contact resistance and other characteristic parameters. The sputtering conditions of the active layer used in the experiment were as follows: the sputtering power was 80 W, sputtering pressure was 5 mTorr, and sputtering oxygen concentration was 5%. The thickness of the NdIZO films was targeted at 15 nm by controlling the sputtering time. The films were annealed at the temperature of 25 °C, 250 °C, 300 °C, 350 °C, and 400 °C for 1 h in air after sputtering the active layer. The sputtering conditions of the S/D electrodes selected in the experiment were: the sputtering power was 100 W, the sputtering pressure was 3 mTorr and the sputtering atmosphere was pure argon. Cu film with a thickness of 300 nm was sputtered under the above conditions.

**Figure 7:**
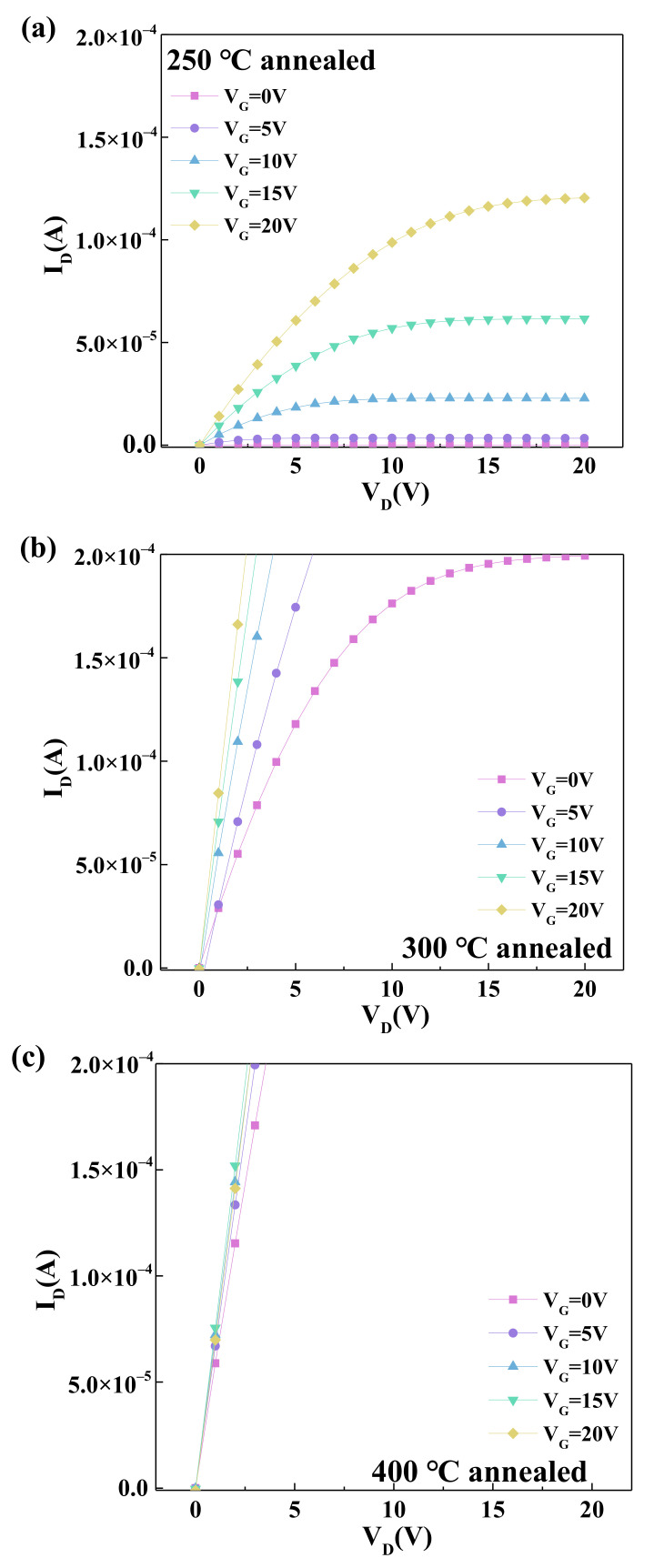
The output characteristics of the NdIZO TFTs at annealing temperatures of (**a**) 250 °C, (**b**) 300 °C, and (**c**) 400 °C.

The NdIZO thin film transistor has nearly no conductivity when it is not annealed. Figure 7 shows the output curves when the annealing temperatures are 250 °C, 300 °C, and 400 °C, respectively. The NdIZO TFT that was annealed at 250 °C has a plump curve shape and exhibits good output characteristics. The device with an annealing temperature of 300 °C has a “too plump” curve. As V_G_ increases, a linear relationship can be found between I_D_ and V_D_. When the annealing temperature reaches 400 °C, I_D_ and V_D_ have a completely a linear relationship, and the output characteristic curve is consistent with that of the resistance.

**Figure 8:**
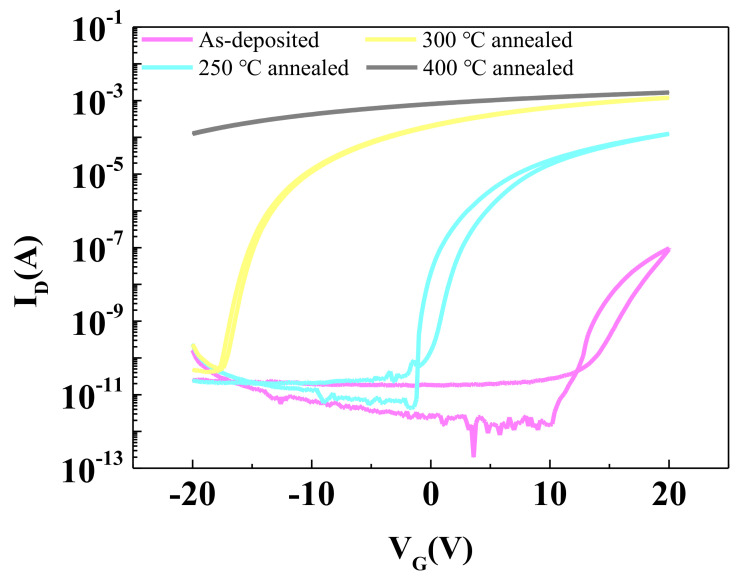
The transfer characteristics of the NdIZO TFTs at different annealing temperatures.

The transfer characteristics of the NdIZO TFTs when as-deposited and the annealing temperatures are 250 °C, 300 °C, and 400 °C are shown in Figure 8. The NdIZO TFT had a weak field effect when it was not annealed. The I_D_ in the open positions was too small, which was about 10^−7^ orders of magnitude. When the annealing temperature rose to 250 °C, the NdIZO TFT had switching characteristics, the I_on_/I_off_ ratio reached 2.89 × 10^7^, the μ_sat_ was around 24.48 cm^2^/(V·s), the SS is 1.14 × 10^−1^ V/decade and the V_G_ was close to 0 V (2.32 V), as shown in Table 2. When the annealing temperature was 300 °C, the I_on_/I_off_ ratio was essentially flat, but there was a significant rise for μ_sat_ while there was a decrease for SS, which could be attributed to an increase in defect states, especially oxygen vacancies. Bonds will break after obtaining enough energy, resulting in numerous defects. The defect state captures certain carriers, which means they no longer participate in conduction. So, the decline in SS was predictable. In order to explain the increase in electrical conductivity, we assumed that there exists a special class of defects among those caused by the annealing temperature rising, such as oxygen vacancy. Oxygen vacancy is a donor defect and provides excess charge carriers that cannot be controlled by the gate voltage. The rise in annealing temperature and fracture of the M-O bond push the balance of Equation (4) to move in the positive direction.

O^x^_2_ → O_2_↑ + V_O_^2+^ + 2e^−^ (4)

**Figure 9:**
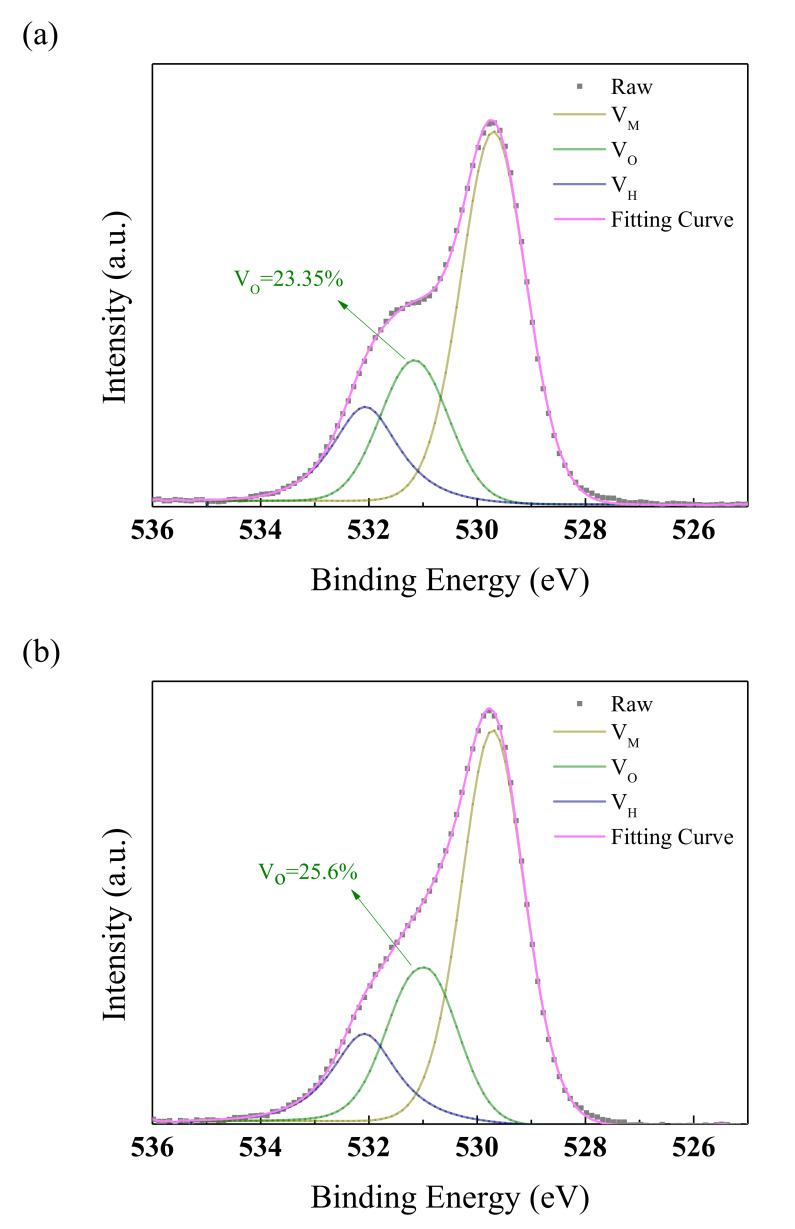
The O1s core level spectra of the NdIZO films with different annealing temperatures: (**a**) 250 °C, (**b**) 300 °C.

An XPS measurement was taken to test the hypothesis, as seen in Figure 9. In oxide semiconductors, oxygen vacancies will provide excess carriers that cannot be controlled by the gate voltage [33]. When the annealing temperature continues to rise, the excess carrier concentration gradually outnumbers the carrier concentration driven by the gate voltage in quantity, making the I_D_-V_G_ image an almost horizontal line, and the device loses its switching characteristics.

In order to further characterize the effect of the annealing temperature on the electrical properties of NdIZO-TFTs, the transmission line method (TLM) [34] was used to evaluate the contact performance of the device (Equation (5)). TLM utilizes a simple series resistant model that describes channel resistance increases with increasing channel length while the contact resistance between the channel and metallization remains the same [35,36].

R_total_ = V_DS_/I_DS_ = r_ch_L + R_C_ (5)

R_total_ refers to the total resistance, L the channel length, r_ch_ the channel resistance per unit channel length, and R_C_ the contact resistance. By preparing thin film transistors with the same channel width and different channel lengths, measuring the transfer curve of each channel length when V_D_ = 0.1 V, the R_total_ could finally be calculated. Then, taking the R_total_ as the *y*-axis and the L as the *x*-axis, the relationship between R_total_ and L was linearly fitted. The slope of the fitted straight line is r_ch_ and the intersection on the *y*-axis is the R_C_.

**Figure 10:**
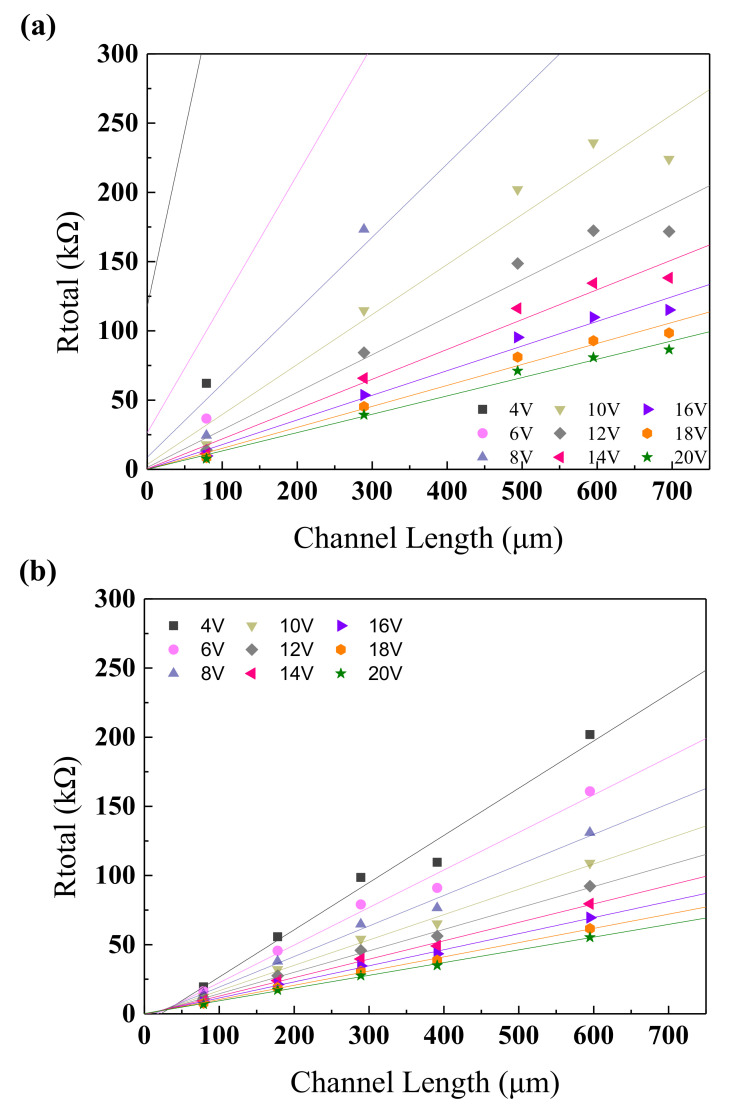
Function images of R_total_ and L at annealing temperature of (**a**) 250 °C and (**b**) 300 °C.

Figure 10 shows the fitting images when the annealing temperature is 250 °C and 300 °C. When the annealing temperature is 250 °C, the fitted lines do not intersect at one point, and each intercept of the ordinate represents the R_C_ under a specific V_G_. However, when the annealing temperature is 300 °C, the result is just the opposite, that is, the fitted lines intersect at one point and the R_C_ becomes a constant value.

**Figure 11:**
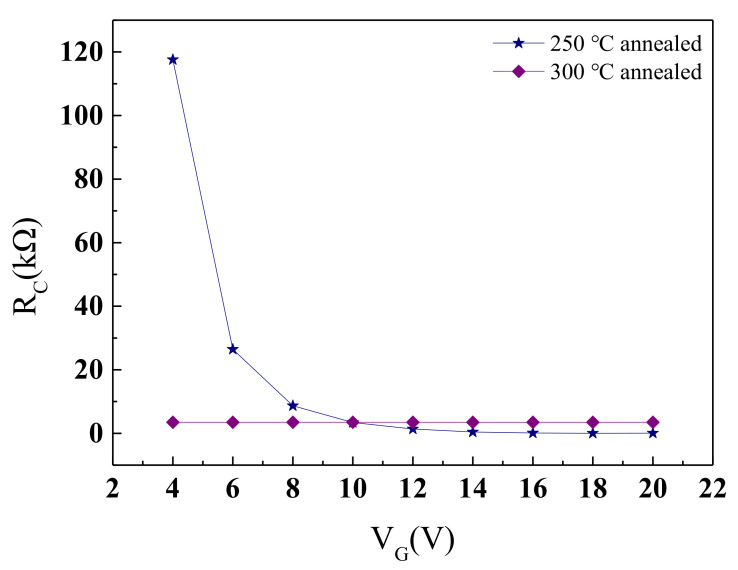
Function images of R_C_ and V_G_ at annealing temperatures of 250 °C and 300 °C.

Figure 11 shows the relationship between R_C_ and V_G_ when the annealing temperature is 250 °C and 300 °C. Under the annealing temperature of 250 °C, as the V_G_ rises from 4 V to 20 V, its R_C_ decreases from a relatively large 117.56 kΩ to around 61 Ω. Low V_G_ corresponds to high R_C_ and high V_G_ corresponds to low R_C_, which realizes the modulation of V_G_ on R_C_ and suggests that the NdIZO-TFTs have dynamic contact characteristics. When the annealing temperature is 300 °C, after extracting the intersection of the fitted lines at different V_G_s, a fixed value of 3.47 kΩ was obtained, which is similar to the contact situation of a highly doped device [37,38,39,40,41,42,43]. This also confirms the possibility that the carrier concentration increases with the increase in the annealing temperature.

**Figure 12:**
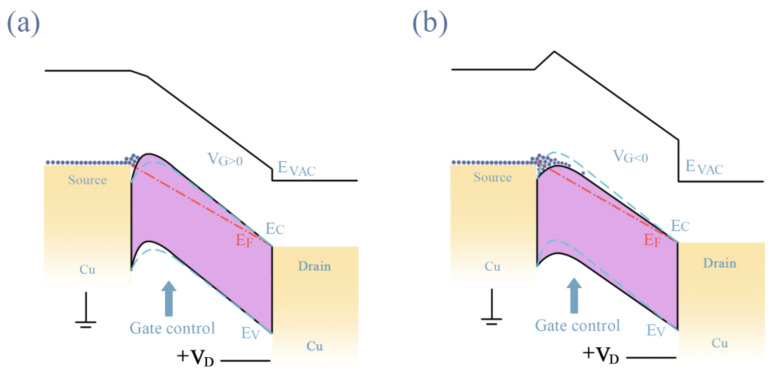
Schematic diagram of the energy band for carrier transportation from source to drain at annealing temperature of (**a**) 250 °C and (**b**) 300 °C.

Finally, a hypothetical band diagram of the contact between the Cu S/D electrode and NdIZO semiconductor film at 250 °C and 300 °C is shown in Figure 12, based on the one proposed by Lu et al. [44]. Different electrical conductivity at different temperatures leads to different contact barriers. As shown in Figure 12a, at 250 °C, the carrier needs to tunnel through an additional block layer, which is equivalent to increasing the potential barrier of the conduction band. Thus, an enhanced mode with a normally closed channel was implemented, since a positive gate voltage is required to conduct the channel. As shown in Figure 12b, the end of the energy band is significantly bent with an annealing temperature of 300 °C, and a potential well will be formed through the metal–semiconductor contact effect, resulting in the failure to close at V_G_ = 0 V. A very negative gate voltage is needed to exhaust the remaining free electrons, which is equivalent to increasing the potential barrier of the conduction band.

## 4. Conclusions

In summary, this study developed a thin film transistor with a NdIZO active layer. When the semiconductor layer is sputtered under conditions of 80 W, 5 mTorr, and 5% of oxygen concentration, the thin film transistor exhibits good uniformity. An increase in annealing temperature leads to the dissociation of oxygen, which leads to an increase in oxygen vacancy, resulting in an increase in carrier concentration, interface defect states and SS. The contact resistance generally changes from high resistance and is controlled by gate voltage, and then finally, it changes to a constant value with the increase in annealing temperature and shows contact characteristics similar to heavily doped devices at 300 °C. Finally, this paper found that the NdIZO TFT with a thickness of about 15 nm after annealing at 250 °C shows good electrical characteristics with an I_on_/I_off_ ratio of 2.89 × 10^7^, a saturation mobility of 24.48 cm^2^/(V·s), and a threshold voltage of 2.32 V. The contact resistance is controlled by the gate voltage, and decreases with the increase in the gate voltage, exhibiting dynamic contact characteristics. Further, the relatively low preparation temperature also facilitates the manufacture of flexible devices.

## Figures and Tables

**Table 1:** Summary of NdIZO film properties in different deposition conditions (at a uniform sputtering power of 80 W).

Oxygen Concentration	Pressure/mTorr	Annealing/°C	Peak Value/mV	τ_2_/μs
0%	3.38	25	540.63	3.57
5%	5.00	25	570.13	3.25
10%	8.00	25	571.04	2.57
0%	3.38	200	539.58	3.28
5%	5.00	200	566.06	2.15
10%	8.00	200	549.94	2.04
0%	5.00	250	577.27	1.75
5%	8.00	250	513.69	2.16
10%	3.38	250	520.87	1.27
0%	8.00	300	467.21	2.66
5%	3.38	300	505.42	0.99
10%	5.00	300	490.55	1.45
0%	5.00	350	509.45	3.45
5%	8.00	350	536.48	3.87
10%	3.38	350	560.83	2.18
0%	8.00	400	44.06	3.47
5%	3.38	400	544.87	2.97
10%	5.00	400	482.20	2.04

**Table 2:** Electrical performance parameters of NdIZO TFTs at different annealing temperatures (* indicates that the value does not exist).

Annealing Temperature (°C)	I_on_/I_off_	μ_sat_ (cm^2^/(V·s))	SS (V/decade)	V_th_ (V)
As-deposited	*	*	*	*
250	2.89 × 10^7^	24.48	1.14 × 10^−1^	2.32
300	2.39 × 10^7^	38.90	5.96 × 10^−1^	−21.52
400	*	*	*	*

## Data Availability

Data is contained within the article.
